# Probiotic-Based Vaccines May Provide Effective Protection against COVID-19 Acute Respiratory Disease

**DOI:** 10.3390/vaccines9050466

**Published:** 2021-05-06

**Authors:** Sedigheh Taghinezhad-S, Amir Hossein Mohseni, Luis G. Bermúdez-Humarán, Vincenzo Casolaro, Naima G. Cortes-Perez, Hossein Keyvani, Jesus Simal-Gandara

**Affiliations:** 1 Department of Microbiology, Faculty of Basic Sciences, Science and Research Branch, Islamic Azad University, Tehran 1477893855, Iran; taghinezhad.m@gmail.com; 2 Université Paris-Saclay, INRAE, AgroParisTech, Micalis Institute, 78350 Jouy-en-Josas, France; luis.bermudez@inrae.fr; 3 Department of Medicine, Surgery and Dentistry “Scuola Medica Salernitana”, University of Salerno, Baronissi, 84081 Salerno, Italy; vcasolaro@unisa.it; 4 Université Paris-Saclay, INRAE, AgroParisTech, UMR 0496, 78350 Jouy-en-Josas, France; naima.cortes-perez@inrae.fr; 5 Department of Virology, Faculty of Medicine, Iran University of Medical Sciences, Tehran 1449614535, Iran; keyvani.h@iums.ac.ir; 6 Nutrition and Bromatology Group, Department of Analytical Chemistry and Food Science, Faculty of Food Science and Technology, University of Vigo—Ourense Campus, E32004 Ourense, Spain; jsimal@uvigo.es

**Keywords:** coronavirus, SARS-CoV-2, COVID-19, probiotics, vaccines, mucosal immunization

## Abstract

Severe acute respiratory syndrome coronavirus 2 virus (SARS-CoV-2) infection, the causative agent of COVID-19, now represents the sixth Public Health Emergency of International Concern (PHEIC)—as declared by the World Health Organization (WHO) since 2009. Considering that SARS-CoV-2 is mainly transmitted via the mucosal route, a therapy administered by this same route may represent a desirable approach to fight SARS-CoV-2 infection. It is now widely accepted that genetically modified microorganisms, including probiotics, represent attractive vehicles for oral or nasal mucosal delivery of therapeutic molecules. Previous studies have shown that the mucosal administration of therapeutic molecules is able to induce an immune response mediated by specific serum IgG and mucosal IgA antibodies along with mucosal cell-mediated immune responses, which effectively concur to neutralize and eradicate infections. Therefore, advances in the modulation of mucosal immune responses, and in particular the use of probiotics as live delivery vectors, may encourage prospective studies to assess the effectiveness of genetically modified probiotics for SARS-CoV-2 infection. Emerging trends in the ever-progressing field of vaccine development re-emphasize the contribution of adjuvants, along with optimization of codon usage (when designing a synthetic gene), expression level, and inoculation dose to elicit specific and potent protective immune responses. In this review, we will highlight the existing pre-clinical and clinical information on the use of genetically modified microorganisms in control strategies against respiratory and non-respiratory viruses. In addition, we will discuss some controversial aspects of the use of genetically modified probiotics in modulating the cross-talk between mucosal delivery of therapeutics and immune system modulation.

## 1. Introduction

The end of 2019 was marked with the beginning of the COVID-19 outbreak caused by the Severe Acute Respiratory Syndrome Coronavirus 2 (SARS-CoV-2) [1]. As of March 2021, more than 115 million cases and 2.5 million SARS-CoV-2-associated deaths have been reported worldwide (WHO COVID-19 Disease Dashboard, 2020). SARS-CoV-2 is a positive-sense, single stranded RNA virus that replicates in the cytoplasm and encodes several structural and non-structural proteins with a genome size of around 29.9 kb (accession code MN908947) [2]. The high rates of SARS-CoV-2 transmission between humans [3], and the current lack of fast-paced, comprehensive vaccination strategies to contain the spread of this infection, make this pandemic a major international public health concern [4]. Therefore, studies are ongoing to find effective drugs to treat and prevent COVID-19. It is generally accepted that vaccination is the most effective approach to control the spread of SARS-CoV-2 transmission [5].

**Figure 1:**
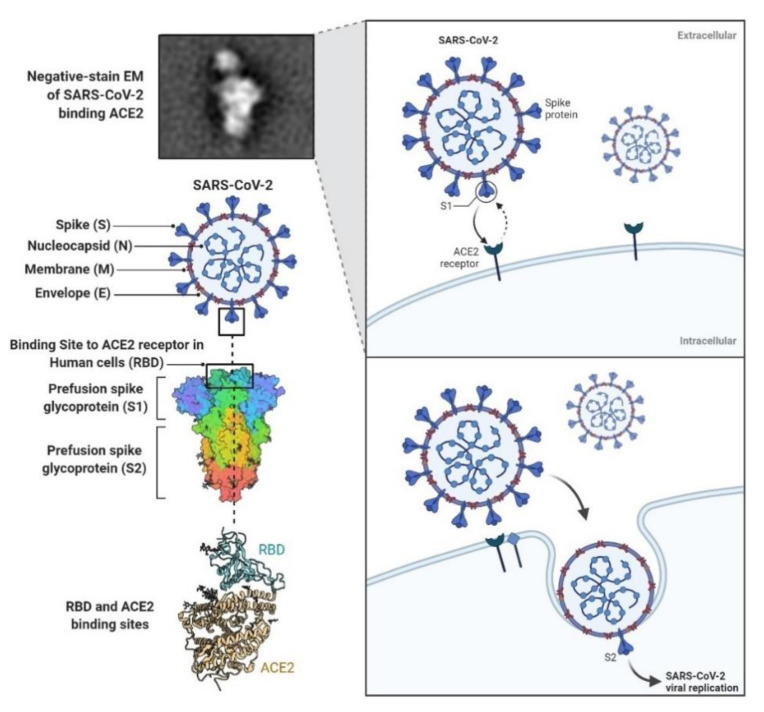
Schematic representation of the structure of important SARS-CoV-2 proteins, targeting the ACE2 receptors and promoting viral entry in infected cells. The SARS-CoV-2 spike (S) protein mediates membrane fusion by binding to these cellular receptors (retrieved from https://app.biorender.com/biorender-templates (access date: 6 March 2021)): *“SARS-CoV-2 Targeting of ACE2 Receptor and Entry in Infected Cell”*).

The four major structural proteins of SARS-COV-2 are the spike (S) glycoprotein, and the nucleocapsid (N), membrane (M) and envelope (E) proteins [6]. The S protein is responsible for attachment to the host cell following recognition of the human angiotensin-converting enzyme 2 (hACE2) receptor, which plays a pivotal role in provoking the immune response during the progression of disease and is targeted by host neutralizing antibodies (Figure 1) [7,8]. Therefore, the S protein serves as a key target in the assessment of SARS-CoV-2-reactive IgG antibodies, as well as the development of COVID-19 vaccines. Additional studies have identified a number of CD4^+^ and CD8^+^ T-cell epitopes within the amino acid sequences of the S protein, stressing their potential roles in inducing T-cell responses [9,10,11]. In addition, current studies suggest that the N protein of SARS-CoV-2 is also a suitable candidate for vaccine development given its high potential to trigger both a humoral and a T-cell immune response in humans [12,13]. 

Unlike the S and N proteins, the M and E proteins are poorly immunogenic and are not targeted by immune responses against coronaviruses, possibly owed to their small ectodomains and small overall molecular sizes [14], as was demonstrated in animal models adoptively transferred with sera from donors vaccinated with a virus vector delivering these proteins [15].

The essential roles of the upper respiratory and/or gastrointestinal tracts as the main routes of SARS-CoV-2 transmission in humans strongly suggest that mucosal delivery of SARS-CoV-2 antigens may represent an attractive and effective strategy for the development of a mucosal vaccine to control COVID-19. Lactic acid bacteria (LAB) are a group of Gram-positive bacteria widely used in industrial food fermentation processes. The most studied genera belonging to LAB are *Lactobacillus, Lactococcus, Streptococcus, Enterococcus*, and *Pediococcus* [16]. Thus, these microorganisms are Generally Recognized As Safe by health authorities, earning a GRAS status by the FDA (USA) [17] and a qualified presumption of safety (QPS) by the EFSA (Europe) [18]. When these microorganisms are ingested live in adequate amounts, they can survive in the host digestive tract, where they are likely to exert various beneficial actions on the host, an effect known as *probiotic* [19,20]. In addition, some genera, such as lactobacilli, are commensal bacteria and considered thus as part of the intestinal microbiota [21]. 

The ability of these microorganisms to survive and colonize the host mucosal surfaces and their immunomodulatory capabilities (i.e., probiotic effects) make them promising vehicles for the delivery of heterologous antigens via mucosal routes [22,23,24,25]. In addition, these vectors, which are easier and safer to administer and cheaper to produce than traditional, injectable vaccines, could be well suited to mass vaccination campaigns in developing countries [26,27,28]. Over the last two decades, research on the use of LAB as live vectors in the development of mucosal vaccines has focused on the construction of genetically modified (or recombinant), strains of the species: *Lactobacillus plantarum*, *Lactobacillus casei*, *Lactobacillus acidophilus*, *Lactobacillus delbrueckii,* and *Lactococcus lactis* able to produce numerous respiratory and non-respiratory virus-derived antigens (among others) (Table 1). Based on these pioneering studies, we can envisage that recombinant LAB-based vaccines may be an attractive option to deliver SARS-CoV-2 antigens to mucosal surfaces and evoke a protective immune response. However, despite numerous reports on the use of recombinant LAB and their demonstrated potential in inducing mucosal immune responses and the prevention of respiratory viral infections, to our knowledge no studies to date have explored the use of recombinant probiotics in the development of vaccines to treat SARS-CoV-2 infections and help control the COVID-19 outbreak. Certainly, more research is needed to demonstrate the full potential of recombinant probiotics. Here, we will discuss the potential antiviral efficiency of a recombinant probiotics-based vaccine, with a special emphasis on SARS-CoV-2 infection. Our goal is to provide a global overview on the use of recombinant LAB in vaccine development, which includes a full understanding of the mechanisms involved in the crosstalk between mucosally delivered therapeutics and the host immune system. This knowledge will be key in the design of future in vitro and preclinical studies as well as subsequent clinical trials. 

## 2. Recombinant Probiotics as Inducers of Humoral Immune Responses

Mucosal vaccination is advocated in several extensive studies as one of the most effective approaches to control and prevent respiratory viral infections [28,29,30]. In view of the fact that the mucosal surfaces of the respiratory tract are the major portal of entry and initiation of respiratory viral infections, it would be desirable to design a vaccine able to elicit specific functions of the mucosal immune system, such as the production of secretory IgA (sIgA) dimers. sIgA are a critical component of mucosal immunity in the respiratory tract, in that they can inhibit the entrance and proliferation of respiratory viruses in the airway mucosa [69,70]. In fact, detailed and in-depth research studies found that sIgA play a more critical role than IgG antibodies in the prevention of viral infections in the upper respiratory tract, including the nose and trachea, due to their ability to decrease virus attachment and avoid virus internalization at the mucosal surfaces [31,71,72]. Besides their role in the prevention of infection, recent work also suggests a putative role of sIgA in the maintenance of microbiota homeostasis [73]. Therefore, stimulation of mucosal immunity has received particular attention in the development of strategies to fight pathogenic microorganisms. Among the different formulations for mucosal immunization, genetically modified LAB have been explored as effective vehicles for antigen delivery due to their safety. Among these, *L. lactis*, *L. plantarum*, and *L. casei* have received special attention due to their superior effects relative to other LAB strains in comparative studies [32,33,74,75,76]. 

Several *L. plantarum*-based model vaccines against viral disease have been constructed and tested in animal studies, such as *L. plantarum* carrying the hemagglutinin–neuraminidase protein (HN) of Newcastle disease virus (NDV) [31], the hemagglutinin (HA) gene of H9N2 avian influenza virus (AIV) [28,34], and different proteins of influenza virus [33,35,36], all of which effectively inducing measures of mucosal immunity (sIgA) as well as serum IgG antibody responses, indicating an encouraging gut-lung axis for orally administered vaccines to combat respiratory viral infections. 

More recently, *L. lactis* was also extensively used to develop various oral-based mucosal vaccines. *In vivo* studies showed that oral administration of *L. lactis* displaying various viral antigens can stimulate robust mucosal and systemic immunity. In addition, several studies have demonstrated that oral immunization with a recombinant probiotic could result in the secretion of sIgA at sites besides the gastrointestinal tract, as these were detected in bronchoalveolar lavage fluids (BALF), ophthalmic and vaginal washings, consistent with acquired resistance to respiratory, gastrointestinal, and genital tract infections [22,29,77,78].

Subsequent studies focused on the induction of mucosal immune responses by another probiotic, *L. casei*. A surface antigen display system was designed using anchoring matrix such as poly-γ-glutamate synthetase A (pgsA) protein of *Bacillus subtilis*, which could effectively express different viral antigens at the surface of *L. casei*, including conserved matrix protein 2 of divergent influenza subtypes [37], HA2 and sM2 influenza antigens [38], N protein of TGEV [39], and HPV-16 L2 protein [40]. Oral and/or nasal administration of these recombinant *L. casei* preparations in mice resulted in stronger induction of serum IgG as well as sIgA against the displayed antigens. Similar results were observed in mice and other animal models following oral administration of recombinant *L. casei* harboring major protective antigen VP4 of porcine rotavirus [23], recombinant protein of TGEV [41], or co-expressing epitopes of porcine parvovirus (PPV) and classical swine fever virus (CSFV) [42], indicating an efficient induction of protective immunity against various viral infections. In light of the valuable insights provided by these studies with genetically engineered *L. casei* carrying viral antigens, more in vivo studies focused on the expression of VP2 protein from infectious pancreatic necrosis virus (IPNV) in recombinant *L. casei*, which resulted in the stimulation of systemic and local mucosal immune responses, high-level production of IgM and IgT, and reduction of viral load in orally immunized rainbow trouts [26,43]. 

**Figure 2:**
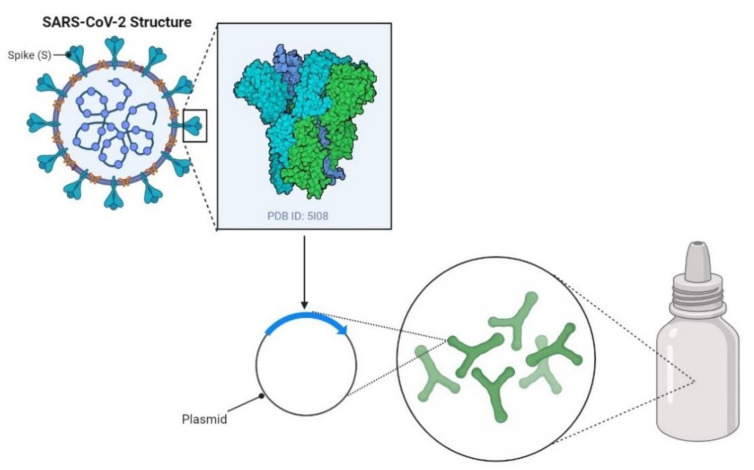
Diagram illustrating the development of the first oral COVID-19 vaccine candidate based on recombinant *Bifidobacterium longum* which has been engineered to deliver plasmids containing synthetic DNA encoding spike protein from SARS-CoV-2 (adapted from https://app.biorender.com/biorender-templates (access date: 6 March 2021)).

Three human clinical trials in support of these pre-clinical findings were launched, in which oral immunization of recombinant *L. lactis* and *L. casei* carrying HPV-16 antigens induced high levels of specific serum IgG and vaginal IgA in volunteers who completed the vaccination schedules [27,44,45]. Interestingly, oral administration in mice of *L. acidophilus* carrying Gag antigen from human immunodeficiency virus 1 (HIV-1) [46] or protein HA1 from highly pathogenic avian influenza (HPAI) virus (H5N1) [22] could only stimulate local sIgA production in the digestive tract, while expression of protein HA1 of HPAI virus by recombinant *L. delbrueckii* subsp. *lactis* could provoke a mucosal immune response in both the gastrointestinal and the respiratory tract [22]. The first-in-human study of an orally delivered probiotic-based SARS-CoV-2 vaccine, called bacTRL-Spike-1, has been designed by Symvivo Corp. (Melbourne, Victoria, Australia), which makes use of engineered *Bifidobacterium longum* to deliver plasmids harboring a full-length S protein gene (Figure 2). To functionally characterize the safety, tolerability, and immunogenicity of the vaccine for the prevention of COVID-19 in healthy adults, three different oral doses of live recombinant *Bifidobacterium longum*, 1 billion (Group 1), 3 billion (Group 2) or 10 billion (Group 3) colony-forming-units (cfu), will be evaluated in subjects 18 years of age and older during a Phase 1, randomized, observer-blind, placebo-controlled trial (NCT number: NCT04334980). The final results of this trial will be made available on 28 February 2022.

## 3. Recombinant Probiotics as Inducers of Cell-Mediated Immune Responses

In recent years, it has become clear that studies aimed at addressing the induction of T cell-mediated immune responses combined to the humoral immune response could offer a much broader scope of protection against invading pathogens such as respiratory viruses. Major progress has been made to define the potential of recombinant probiotic vaccines in the stimulation of T cell-mediated immune responses in addition to IgG and sIgA production, which, combined, could open up new opportunities in the fight against viral infection [47,79,80]. Therefore, uncovering the ability of recombinant probiotics to elicit T cell-mediated immune responses of proper sign and intensity might help predict their likely impact on the ongoing COVID-19 pandemic.

### 3.1. T Helper

**Figure 3:**
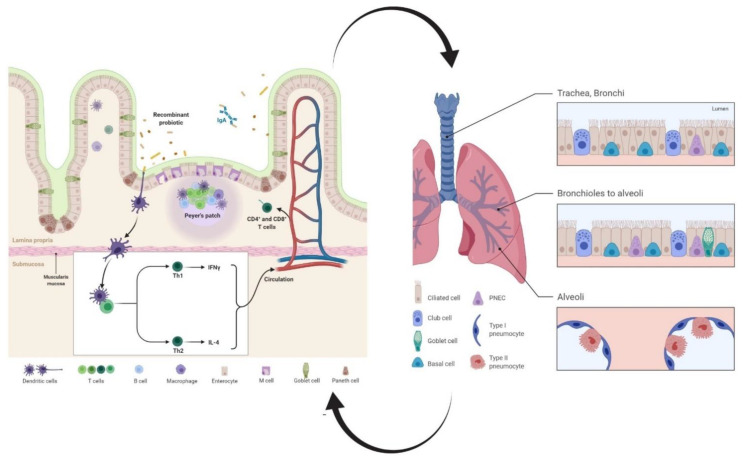
Schematic representation of the stimulation of immune responses with genetically modified probiotics expressing therapeutic factors in the gut and the lung. The crosstalk and the reciprocal interaction of the gut and lung mucosa (gut-lung axis) is mediated by immune cells moving between the two districts via the bloodstream and the lymphatic ducts, leading to modulation of the immune response in both sites. Delivery of antigen via recombinant probiotic to antigen-presenting cells in Peyer’s patches causes the stimulation of naive B and T cells and induction of several immune factors, such as Th1 and Th2 cytokines. As a result, cells and immune factors migrate to the thoracic duct and the BALT through circulation and enhance the production of secretory IgA and the activation of effector CD4^+^ and CD8^+^ T cells, preventing the onset and progression of respiratory viral infections. IgA, immunoglobulin A, IL-4: interleukin-4, IFN-γ: interferon-gamma, Th1: T-helper cell type 1, Th2: T-helper cell type 2, BALT: bronchi-associated lymphoid tissue (adapted from https://app.biorender.com/biorender-templates (access date: 24 November 2020).

CD4^+^ T cells exposed to diverse pathogens can express diverging effector phenotypes, the best studied of which are characterized by predominant production of the cytokines, interferon (IFN)-γ or interleukin (IL)-4 and have been termed T helper (Th) 1 and Th2 cells, respectively. Substantial production of Th2-type (IL-4) and Th1-type (IFN-γ) cytokines was reported in response to mucosal administration of *L. casei* and *L. lactis* carrying viral antigens in immunized mice, variably contributing to host defense against viral infection [37,48,49,50]. In agreement with earlier studies, oral vaccination of mice with recombinant *Lactobacillus* strains and *L. lactis* expressing viral antigens could induce IL-4 production and provoke a proliferative response of splenic lymphocytes, raising the possibility that common mucosal immunization might stimulate Th2-like cell-mediated immunity [22,51,52]. However, it is well known that IFN-γ, produced by NK cells and T lymphocytes, and tumor necrosis factor (TNF)-α, produced by T lymphocytes and monocytes, play major roles in antiviral immunity. It has long been known that IFN-γ plays an important role in promoting phagocytic activity against viral and bacterial infection and TNF-α is a major mediator of inflammatory responses [34,81]. Production of TNF-α and IFN-γ by Th1 cells has a significant role in the activation antiviral responses via stimulating macrophages and cells associated with cell-mediated cytotoxicity [36,82]. In fact, IFN-γ and TNF-α levels could significantly increase in mice receiving *L. plantarum*, *L. casei*, and *L. lactis* expressing viral genes, implying that recombinant probiotics could modulate adaptive immunity by up-regulating the effector responses of CD8^+^ T cytotoxic cells and CD4^+^ Th cells [27,34,48]. Consistent with this, in vivo administration of recombinant *L. plantarum* expressing influenza virus H9N2 protein resulted in the generation of protective immune responses through the expansion of IFN-γ-expressing CD8^+^ T cells and Th1 cells [28,34,36] (Figure 3). 

### 3.2. T Killer

Studies in mice orally administered with *L. lactis* expressing HPV-16 antigens showed the expansion of specific IL-2-secreting CD4^+^ T cells and IFN-γ-secreting CD8^+^ T cells in the intestinal mucosa, and of vaginal and splenic lymphocytes, resulting in protective and therapeutic anti-tumoral responses against challenge with an E6/E7-expressing tumor cell line (TC-1) [32,53]. Recent findings also show that expansion of IFN-γ-secreting CD4^+^ and CD8^+^ T cells, and stimulation of mucosal Th1 immune responses by recombinant *L. casei* could elicit confer a substantial level of protection against viral infections in humans [44,54]. Clinical studies found that oral vaccination with recombinant *L. lactis* containing viral antigens could stimulate production of high amounts of IFN-γ at the intestinal mucosal inductive sites (Peyer’s patches). By contrast, the poor ability of recombinant *L. lactis* to induce systemic immunity has been documented elsewhere [27,45]. Together, these observations suggest that mucosal T cells stimulated by recombinant *L. lactis* in the gut, while initially moving to the peripheral circulation, will eventually home and settle in the specific mucosa. In any instance, these studies strongly point to the role of CD8^+^ cytotoxic T lymphocytes (CTLs) induced by recombinant probiotics in promoting viral clearance. For instance, recombinant *L. casei* can efficiently stimulate CSFV-specific CD8^+^ CTL responses to protect pigs against CSFV challenge [42]. This notion is further confirmed in studies showing that recombinant *L. plantarum* could provoke the expansion of CD8^+^ CTLs conferring protection and increased survival against lethal influenza virus challenge [36]. Additionally, we found preclinical evidence that recombinant *L. casei* and *L. lactis* expressing viral antigens could sustain long-lasting immune responses, which were observed at least 2–6 months after the last vaccine boost [37,38,55]. These data are supported by clinical evidence showing long-term specific CTL responses against HPV-16 during 6-month follow-ups in healthy females, further demonstrating that recombinant LAB can elicit long-lasting immunity against viral pathogens [27,45].

### 3.3. Dendritic Cells (DCs)

Dendritic cells (DCs) were characterized as the bridge between host innate and adaptive immunity. DCs can efficiently trap self and foreign antigens and present them to naïve T cells in secondary lymphoid tissues. DCs isolated from mucosal tissues and the spleen could favorably stimulate Th2 and Th1 responses, respectively [34,83]. The potential role of DCs in promoting strong cellular immunity toward genetically modified *L. lactis* and *L. plantarum*-derived antigens was shown in some in vivo studies. Consistent with this, it was long believed that the oral administration of these strains could induce Peyer’s patch (PP) DCs activation [33,56]. Compelling evidence in animal studies shows DC stimulation in the small intestine mucosa and the mesenteric lymph nodes (MLNs) by probiotic strains, which would support pathogen’s killing. Owing to the distinct ability of DCs to elicit an immune response, DC targeting strategies have received more attention in vaccinology. A specific DC-targeting peptide (DCpep) was utilized in some studies to enhance the robustness of immune responses [31,36]. For example, Wang et al. fused the S gene of SARS-CoV-2 with DCpep and reported the successful expression of recombinant S protein on the surface of *L. plantarum* [57]. As a result, a significant increase in the percentages of CD4^+^ T cells was observed in the spleen and peripheral blood of mouse and chicken models after immunization with *L. plantarum* expressing DCpep fused with viral antigens. In contrast, such a response was not observed in animals immunized with recombinant *L. plantarum* not expressing DCpep. Along the same line, the few in vivo studies conducted to date showed that *L. plantarum* expressing viral antigens attached to DCpep could effectively stimulate DCs activation in PPs, MLNs, and the small intestine. These findings indicate the potential usefulness of DCpep fusion antigens to provide an effective immune adjuvant in the development of a mucosal vaccine [36,58,84]. Building on these studies, Jiang et al. have provided evidence of an association between DC activation and the promotion of T-cell differentiation, both contributing to pathogen clearance in animal models [31]. Moreover, probiotics-based vaccines can regulate the elicited immune responses by interacting with Toll-like receptors (TLRs) on macrophages and DCs [85]. As well, one in vivo study indicated that inhibiting TLR expression could be achieved by a *Lactobacillus*-vaccine; thus neutralization of viruses will occur [41].

## 4. Optimization of the Immune Response Induced by Recombinant Probiotic-Based Vaccines

Studies performed to date show that heterologous proteins in some recombinant probiotics may be expressed at low levels, likely due to the intrinsic low-copy number of a shuttle vector. Given this limitation, the appearance of weak signals in the subsequent IFA tests and/or Western blot experiments would be the main drawback of recombinant probiotic-based vaccines [24,86]. Attempts have been made to produce higher recombinant protein levels by optimizing nutrients, such as protein or sugar sources, in a fermenter/bioreactor under controlled pH conditions to prevent batch-to-batch variability [87]. In some cases production of recombinant proteins was increased by optimizing the temperature at the induction point to prevent protein degradation in probiotics [48,88,89]. Codon optimization in probiotics such as *L. casei* and *L. lactis* has been shown to be an important factor to optimize the translational efficiency of heterologous proteins and dramatically enhance the overall yield of recombinant proteins [90,91,92]. These measures dramatically reduce the number of non-matching genes containing native codons, which enables to select codons corresponding to those of the probiotic hosts which generate a higher level of recombinant expression of the protein [93]. It is documented that recombinant *L*. *lactis* harboring codon-optimized oncogenes of HPV-16 had an improved inhibitory effect on tumor size progression and tumor growth, thus resulting in better survival rates in vivo compared to those with native codons [32,52]. Using a similar approach, successful expression of influenza virus genes was reported in *L. casei* in vitro [24,91]. These optimized responses were postulated to result from substantial enhancement in humoral and cellular immunity elicited after administration of recombinant probiotics.

Data collected over the past decade indicate that optimizing vaccine dose during dose-escalation studies may be considered one of the most important factors to properly stimulate a mucosal immune response in animals and humans [59,94]. This point was supported in a few clinical studies showing that the number of viable colonies (colony-forming units: CFU) of recombinant probiotics correlates with the efficacy of immune responses. Consistently, Mohseni et al. and Taghinezhad et al., in a Phase I, proof-of-concept clinical trial, showed that the induction of humoral and cell-mediated immune responses in volunteers who received 5,000,000,000 CFU/mL of recombinant *L. lactis* were more robust than in those receiving 1,000,000,000 CFU/mL, a parameter clearly depending on the dose of this strain administered for mucosal immunization [27,45]. Nevertheless, no studies have systematically investigated the impact of dose escalation on the expression of respiratory viral genes in probiotics and the ensuing immune responses to fight off these pathogens. Clearly, additional studies will be needed to confirm this theory. 

This information would also assist greatly in interpreting the effects of specific adjuvants on the robustness of protective responses [35]. Concerning this aspect, different adjuvants have been used to properly enhance the immune response to recombinant probiotics. A number of adjuvants have been used in these studies, including heat-labile toxin B subunit (LTB) [23,42], heat-labile toxin LT (R192G/L211A) [60], FliC [46], CTA1 [38], Gram-positive enhancer matrix (GEM) [95,96], AcmA [29], DC-targeting peptides (DCpep) [31], the nontoxic B subunit of cholera toxin (CTB) [36,50], MDP, and tuftsin [41]. In addition, results from other studies suggest that the provision of definite amounts of IL-2, IL-18, IL-1, and IL-10 as adjuvants may further improve the elicited immune response [97,98]. However, it is essential to emphasize that LAB possess inherent adjuvant characteristics, sufficient to properly induce the host immune system thanks to their intrinsic immunomodulatory properties [34,99,100]. The potential adjuvant effects of LAB could be attributed to the systemic release of specific cytokines which stimulate innate immunity [101,102,103]. According to this paradigm, striking results from in vitro and in vivo studies provide evidence that probiotic strains could exert their adjuvant functions by up-regulating DC and Th1 cytokines and down-regulating Th2 activity [104,105]. However, the exact mechanisms mediating these functions are not fully elucidated.

## 5. Discussion

Experience in the past decades have clearly demonstrated that a shift from traditional needle-based immunization to a needle-free one can overcome a number of limitations, thus accelerating large vaccination programs, particularly in resource-limited developing countries. In particular, studies within this area of investigation have led to discover that mucosal immunization, which entails the delivery of heterologous proteins to mucosal surfaces, is one of the few needle-free approaches that can exert significant prophylactic and therapeutic effects [106,107,108]. Mucosal vaccines have rapidly raised considerable practical and conceptual interest due to their easy administration, low cost, the ability to provoke mucosal, humoral, and systemic immune responses, the negligible risk of blood-borne infections, and the convenient distribution, not requiring a cold chain [109,110,111]. Intensive efforts have been carried out by many groups over the past years to develop mucosal vaccines against an expanding range of pathogens, and their results indicate that delivery of immunogenic molecules to the mucosa via recombinant probiotics administered through nasogastric or orogastric routes is a promising non-invasive way for protection against various infections by improving humoral, mucosal and T-cell-mediated immune responses [44,45,49]. These discoveries sparked a raised level of attention from the scientific community, leading to an ever expanding bulk of studies aimed at defining the best strategies for efficient, high-level expression of heterologous proteins in probiotics to improve the therapeutic effects of probiotic-based vaccines [79,88,89]. Along this line, studies conducted over the last few years have brought substantial insights into the efficacy of prophylactic or therapeutic probiotic-based vaccines against respiratory and non-respiratory viral agents. Vaccines based on LAB, especially *L. plantarum*, *L. casei*, and *L. lactis*, have shown promising beneficial effects, in particular when administered to overcome infections from emerging respiratory viruses, including SARS and influenza viruses [112,113]. 

Increasing evidence indicates that the cellular localization of viral antigens plays a crucial role in the susceptibility of antigens to environmental control and proper recognition by the immune system [32,114]. Heterologous proteins harbored in recombinant probiotics can be expressed in the cytoplasm, anchored to the cell wall, or secreted. It is documented that the expression on the cell wall can generally stimulate more robust host immune responses following immunization with recombinant probiotics compared to preparations resulting in cytoplasmic or secreted expression [115,116]. Several methods exist for anchoring proteins to probiotics, of which the inclusion of a LPXTG anchor motif and poly-γ-glutamic acid synthetase A (*pgsA*) have been the most commonly used for producing viral antigens [30,38,40,61]. Theoretically speaking, exposure of bioactive protein molecules on the surface of probiotics could resist harsh conditions such as proteolysis, improve the antigen’s stability, facilitate antigen presentation, and subsequently provide an effective means for eliciting protective immune response, thus ensuring a higher therapeutic efficacy in challenge experiments than intracellular antigens [116,117]. 

Due to the increasing challenges regarding the safety of probiotic-based vaccines for human health, several studies suggest that a biological containment system represents the best way to prevent the survival of probiotic-based vaccines in the environment outside the host [118,119]. This concept is reinforced by the results obtained in human clinical trials using recombinant probiotic-based vaccines against viruses, which confirm that these vectors have no side effects in humans [27,45,120,121,122]. It is also generally accepted that the use of heat-attenuated probiotic-based vaccines can decrease the spread of antibiotic-resistance genes in humans and in the environment, but this assumption is still awaiting definitive evidence [54,123]. 

Pre-clinical and clinical studies document that among different routes for mucosal immunization, oral immunization offers several advantages, including facilitated stimulation of gut-associated lymphoid tissue (GALT), enhanced production of anti-viral IgA, effective overall induction of mucosal immune responses, decreased risk of contamination, cost-effectiveness, easy self-administration or administration to animals, and antigen access to a larger mucosal area for a prolonged duration [22,23,27,45,50]. It has been speculated that oral vaccination, compared to the nasal route, can significantly increase DC activation, specific sIgA production, CD8^+^ T-cell induction, and cross-protection against viral challenge in vivo [36]. In line with this view, in vivo studies showed that oral intake of recombinant LAB can provide higher neutralizing antibody activities compared to intraperitoneal injection [55,62]. Moreover, oral immunization with recombinant *Lactobacillus* is more effective than the intranasal route in eliciting neutralizing antibodies, including sIgA, in the respiratory tract [124]. It is worth emphasizing that the elicited antibodies in these models exerted potent neutralizing activities against SARS pseudoviruses [30]. By contrast, some of the advantages of intranasal administration relative to oral vaccination would include a reduced frequency of administration, lower inoculation dose, and administration in the same location as the natural infection [106,125]. Using this route to expose immune cells to high concentrations of vaccine would contribute to inhibiting viral colonization in the respiratory tract by effectively inducing sIgA production. In fact, intranasal administration can also induce greater quantities of IgG in serum and of some cytokines in epithelial cells in the lung alveoli than oral administration, thus increasing the speed of the immune and antibody response to viral antigens. These elements may therefore lead to conclude that intranasal inoculation could be a more efficient route for mucosal immunization [35,37,38]. In the end, it is generally accepted that oral immunization could be particularly beneficial for extensive immunization of farm animals where the recombinant probiotic could be administered in drinking water or food. Conversely, since the main infection route of respiratory viruses is nasal, and nasal immunization is more likely to induce high titers of specific antibody titers (mainly sIgA), it is currently reasonable to opt for nasal administration of recombinant probiotics to combat respiratory viral infections. 

In the face of extensive studies in different infection models, still little is known about the potential effect of a probiotic-based vaccine against SARS-CoV-2. However, during the COVID-19 outbreak, all therapeutic options tested against this disease originated mainly from indirect observations and previous knowledge generated in studies of the new influenza A (H1N1) virus, the middle east respiratory syndrome (MERS), and the severe acute respiratory syndrome coronavirus 1 (SARS-CoV-1), among others. Lessons learned from earlier studies of recombinant probiotics to treat other viral infections (both in vitro and in preclinical models), allow us to infer that similar strategies might be devised for the development of a probiotic-based SARS-CoV-2 vaccine. Therefore, it is likely that mucosal immunization, mainly through the nasal route, with a probiotic-based vaccine may strongly inhibit SARS-CoV-2 infection. In conclusion, future efforts are warranted in this area of investigation, in the setup of aptly designed pre-clinical and clinical studies, to explore the potential benefits of mucosal delivery of therapeutics in the fight against COVID-19 pandemic.

## Figures and Tables

**Table 1:** Pre-clinical and clinical studies of probiotic-based vaccines against respiratory and non-respiratory viruses.

Probiotic	Virus	Host/Inoculation Route	Pathways of Immune System Induction	Number	Dosage	Reference
***L. acidophilus***	Avian influenza virus H5N1	Mouse/Oral	Induction of anti-HA1 IgA antibody, anti-HA1 IgG, lymphocyte proliferative reaction, and IL-4	6 times	1 × 10^10^ CFU/mL	[22]
***L. delbrueckii*** **subsp. *lactis***	Avian influenza virus H5N1	Mouse/Oral	Induction of anti-HA1 IgA antibody, anti-HA1 IgG, lymphocyte proliferative reaction, and IL-4	6 times	1 × 10^10^ CFU/mL	
***L. casei***	Porcine rotavirus	Mouse/Oral	Induction of serum IgG and mucosal IgA	9 times	1 × 10^9^ CFU/mL	[23]
	Infectious pancreatic necrosis virus (IPNV)	Rainbow trouts/Oral	Induction of specific IgM anti-pIPNV, and reduction of viral loads	2 times	5 × 10^8^ pfu/200 µL	[26]
***L. lactis***	Human papillomavirus type 16 (HPV-16)	Healthy women/Oral	Induction of E7-specific IgG and SIgA antibody and, E7-specific IFN-γ-secreting CD8^+^ T cell immune response	20 times	1 × 10^9^, 5 × 10^9^, and 1 × 10^10^ CFU/mL	[27]
***L. plantarum***	Influenza virus H9N2	Mouse/Oral	Induction of IgG, sIgA, HI antibodies, and CD8^+^ T cell immune response	7 times	1 × 10^9^ CFU/mouse	[28]
***L. lactis***	Influenza virus H1N1	Mouse/Oral	Induction of specific serum IgG and IgA, and sIgA	9 times	1 × 10^10^ and 5 × 10^10^ CFU/mL	[29]
***L. casei***	Severe acute respiratory syndrome (SARS)	Mouse/Oral and nasal	Induction of serum IgG and mucosal IgA	For oral: 20 times For nasal: 8 times	For oral: 5 × 10^9^ cells/100 µL For nasal: 2 × 10^9^ cells/20 µL	[30]
***L. plantarum***	Newcastle disease virus (NDV)	Chicken/Oral	Induction of sIgA, CD3^+^CD4^+^T, T lymphocytes proliferation and increasing survival rates	9 times	10^9^ CFU/0.2 mL	[31]
***L. lactis***	Human papillomavirus type 16 (HPV-16)	Mouse/Oral	Induction of E7-specific antibody and E7-specific CD4^+^ Th and CD8^+^ T cell precursors, specific IL-2- and IFN-γ-secreting T cells	9 times	1 × 10^8^, 1 × 10^9^, and 1 × 10^10^ CFU/mL	[32]
***L. plantarum***	Influenza A virus H1N1	Mouse/Oral	Induction of Peyer’s patch (PP) DC, PP B220^+^IgA^+^, sIgA, growth centers (GCs) in PPs, T immune response, CD8^+^IFN-γ^+^ cells, and reduction viral load	6 times	-	[33]
	Goose parvovirus (GPV)	Mouse/Oral	Induction of CD11c^+^, CD3^+^CD4^+^, CD3^+^CD8^+^, IFN-γ^+^ and TNF-α, and sIgA	14 times	2 × 10^9^ CFU/mL	[34]
	Avian influenza virus	Chicks/Oral	Induction of specific humoral, mucosal, and T cell-mediated immune responses, and reduction viral load	6 times	2 × 10^9^ CFU/300 μL	[35]
	Avian influenza virus H9N2	Mouse/Oral	Induction of specific mucosal antibody responses and B and T cell responses, specific CD8 T cells, and antigen specific cytotoxicity	6 times	1 × 10^9^ CFU/mouse	[36]
***L. casei***		Mouse/Oral and nasal	Induction of serum IgG, mucosal IgA, and cell-mediated immune response	For oral: 10 times For nasal: 8 times	For oral: 1 × 10^10^ CFU/100 µL For nasal: 1 × 10^9^ CFU/20 µL	[37]
	Influenza A viruses	Mouse/Oral and nasal	Induction of serum IgG and their isotypes (IgG1 & IgG2a), mucosal IgA, sM2- or HA2-specific cell-mediated immunity, IFN-g, and IL-4	For oral: 8 times For nasal: 6 times	For oral: 1 × 10^10^ CFU/100 µL For nasal: 1 × 10^9^ CFU/20 µL	[38]
	Transmissible gastroenteritis virus (TGEV)	Mouse and pregnant sow/Oral and nasal	Induction of IgG and sIgA	For oral :20 times For nasal: 8 times	For oral: 5 × 10^9^ CFU/mL For nasal: 2 × 10^9^ CFU/mL	[39]
	Human papillomavirus type 16 (HPV-16)	Mouse/Oral	Induction of L2-specific serum IgG and vaginal IgG, and IgA	30 times	5 × 10^9^ cells/mL	[40]
	Transmissible gastroenteritis coronavirus (TGEV)	Oral/Piglet	Induction of systemic and mucosal immune responses, cellular immunity, switching from Th1 to Th2-based immune responses	1–48 h	1 × 10^10^ CFU/mL	[41]
	Classical swine fever virus (CSFV) and porcine parvovirus (PPV)	Pig/Oral	Induction of mucosal and systemic CSFV-specific CD8 CTL responses, anti-PPV-VP2 serum IgG, and mucosal IgA	6 times	1 × 10^10^ CFU/mL	[42]
	Infectious pancreatic necrosis virus (IPNV)	Juvenile rainbow trouts/Oral	Induction of IgM and IgT, IL-1β, IL-8, CK6, MHC-II, β-defensin, TNF-1α, and reduction in viral load.	2 times	1 × 10^9^ CFU/mL	[43]
	Human papillomavirus type 16 (HPV-16)	Human/Oral	Induction of E7-specific humoral, cellular, and mucosal immune response	20 times	500, 1000, and 1500 mg/day	[44]
***L. lactis***	Human papillomavirus type 16 (HPV-16)	Healthy women/Oral	Induction of E6-specific IgG and SIgA antibody and, E6-specific IFN-γ-secreting CD8^+^ T cell immune response	20 times	1 × 10^9^, 5 × 10^9^, and 1 × 10^10^ CFU/mL	[45]
***L. acidophilus***	Human immunodeficiency virus 1 (HIV-1)	Mouse/Oral	TLR5-stimulating activity, maturation and cytokine responses of DCs, induction of gamma interferon-producing cells, and Gag-specific IgA-secreting cells	Three daily doses on weeks 0, 2, and 4	2 × 10^9^ CFU/mL	[46]
***L. lactis***	Streptococcus pneumoniae	Mouse/Nasal	Induction of PspA-specific IgG and IgA antibodies, and Th1-mediated immune response	3 times	1 × 10^9^ CFU/mL	[47]
***L. casei***	Porcine epidemic diarrhea virus (PEDV)	Mouse/Oral	Induction of mucosal and systemic immune responses, IL-4, and IFN-γ	9 times	2 × 10^9^ cell/0.1 mL	[48]
***L. lactis***	Avian influenza virus	Mouse/Oral	Induction of specific anti-HA1 IgA and IgG antibodies, IL-4, and IFN-γ	6 times	1 × 10^10^ CFU/mL	[49]
	Avian Influenza (HA1) Virus	Mouse/Oral	Induction of HA-specific serum IgG and fecal IgA, CD8^+^ T cell proliferation, and IFN-γ+	13 times	1 × 10^10^ CFU/mL	[50]
***L. plantarum***	Influenza virus H9N2	Mouse/Oral	Induction of CD3^+^CD4^+^IL-4^+^, CD3^+^CD4+IFN-γ+ and CD3^+^CD4^+^IL-17^+^ T cells, CD3^+^CD8^+^IFN-γ^+^ T cells, serum IFN-γ, IgA, sIgA, and increasing survival rate	9 times	10^9^ CFU/0.1 mL	[51]
***L. lactis***	Hepatitis E virus (HEV)	Mouse/Oral	Induction of ORF2-specific mucosal IgA and serum IgG, and cellular immunity	6 times	1 × 10^10^ CFU/mL	[52]
	Human papillomavirus type 16 (HPV-16)	Mouse/Oral	Induction of specific IgA and IgG, specific IL-2- and IFN-γ-secreting lymphocytes, and increasing survival rate	9 times	1 × 10^9^ CFU/mL	[53]
***L. casei***	Human papillomavirus type 16 (HPV-16)	Human/Oral	Induction of cellular and mucosal immune response	1, 2, 4, or 6 capsules/day at weeks 1, 2, 4, and 8	250 mg/ capsule	[54]
***L. lactis***	Dengue (DEN) virus	Mouse/Oral and nasal	Induction of anti-EDIII antibody responses	6 times	For oral: 1 × 10^10^ CFU/mL For nasal: 1 × 10^8^ CFU/mL	[55]
	Human immunodeficiency virus (HIV)	Mouse/Oral	Induction of HIV-specific serum IgG, fecal IgA, and Cell-mediated immune responses	5 times	1 × 10^8^ CFU/mL	[56]
***L. plantarum***	SARS-CoV-2	-	-	-	-	[57]
	Avian influenza virus H9N2	Mouse and chicken/Oral	Induction of HI antibodies and T cell immune responses	6 times	For mouse:1 × 108 CFU/200 μL For chicken: 5 × 108 CFU/500 μL	[58]
***L. casei***	Human papillomavirus type 16 (HPV-16)	Oral/Mouse	Induction of E7-specific mucosal IFNγ-producing cells and mucosal Th1 immune response	16 times	1 × 10^5^ cells/head	[59]
***L. lactis***	Rotavirus	Mouse/Oral and nasal	Induction of Anti-rotavirus IgG and IgA antibodies, and reduction viral load	For oral: 27 times For nasal: 3 times	30 μg/dose	[60]
	New influenza A H1N1	Mouse/Oral	Induction of anti-HA1 sIgA antibodies and humoral response	9 times	1 × 10^10^ CFU/mL	[61]
	Porcine transmissible gastroenteritis virus (TGEV)	Mouse/Oral	Induction of IgG and IgA antibodies and local mucosal immune responses.	9 times	1 × 10^9^ CFU/mL	[62]
***L. plantarum***	Spring viremia of carp virus (SVCV)	Craps/Oral	Induction of IgM and reduction of viral loads	27 times	1 × 10^9^ CFU/gr	[63]
***L. paracasei***	Rotavirus-induced diarrhea	Mouse/Oral	Reduction of infection in cell cultures, shortened disease duration, severity, and viral load	4 times	1 × 10^7^, 1 × 10^8^, and 1 × 10^9^ CFU/mL	[64]
***L. lactis***	Rotavirus	Mouse/oral	Induction of sIgA and IgG	9 times	1 × 10^9^ CFU/mL	[65]
	Human papillomavirus type 16 (HPV-16)	Mouse/Nasal	Induction of E7-specific cytotoxic T-lymphocyte response, antigen-specific immune response, high survival rate	3 times	1 × 10^9^ CFU/mL	[66]
	Avian influenza virus	Chicken/Nasal	Induction of specific serum IgG	9 times	4 × 10^10^ CFU/100 µL	[67]
*** L. pentosus ***	Transmissible gastroenteritis virus (TGEV)	Mouse/Oral	Induction of serum IgG and mucosal IgA	9 times	2 × 10^9^ CFU/100 µL	[68]
***B. longum***	SARS-CoV-2	Human/Oral	Ongoing project; the final results will be made available on 28 February 2022.	Single dose	1 × 10^9^, 3 × 10^9^, and 10 × 10^9^ CFU	NCT number: NCT04334980

## Data Availability

Not applicable.
